# Orbital pseudotumor

**DOI:** 10.4103/0974-620X.53043

**Published:** 2009

**Authors:** Muqtasid A Kamili, Ali G, Ishrat H Dar, Showkat H Dar, Hardeep Singh Wazir, Tariq Qureishi

**Affiliations:** Department of Medicine, Government Medical College, Srinagar, India; 1 Department of Medicine and Ophthalmology, Government Medical College, Srinagar, India

Orbital pseudotumor also known as Idiopathic Orbital Inflammatory Syndrome (IOIS) is a benign, noninfective inflammatory condition of the orbit without identifiable local or systemic causes. The clinical diagnosis is one of exclusion with evaluation directed to exclude neoplasms, infections, and systemic disorders.[1-3] Orbital pseudotumor was first described in 1903 by Gleason,[4] and characterized as a specific clinicopathological entity in 1905 by Birch-Hirschfeld.[5] It accounts for 4.1−6.3% of orbital disorders and typically occurs in the adult population. The true incidence of orbital pseudotumor is difficult to estimate due to wide spectrum of manifestations and lack of a universally accepted definition of the disease. [6] IOIS is diagnosed by clinical history and evaluation to rule out other causes of orbital disease. Orbital Magnetic Resonance Imaging (MRI) is the single most important diagnostic test, but serological studies and incisional biopsy can be necessary to exclude a systemic cause. [7] Orbital pseudotumor is the third most common orbital disease following Graves ophthalmopathy and lymphoproliferative disease. The immediate response to therapy is generally favorable but relapses are common and persistent inflammation frequently complicates the clinical course.

A 65-year-old Indian male was referred to our hospital with the complaint of progressive bulging of both eyeballs and swelling around the eyes of 10-year duration. The symptoms had apparently started with itching in the left eye, followed by swelling of the lids and gradually progressive forward displacement of the eyeball a few days later, which was accompanied by redness and watering. A month later similar symptoms developed in the right eye. The symptoms progressed over a period of 2−3 years, after which the swelling remained stable. He also complained of progressive blurring progressive and diminution of vision for the last six months. He had no history of fever, painful eye movements, diplopia, headache, previous surgery or trauma. The patient′s past history was negative for systemic hypertension, diabetes or any other medical problems.

General assessment revealed normal vital signs and systemic examination. Eye examination showed bilateral proptosis [Figure 1]; 35 mm in left and 33 mm in the right eye as measured by Hertel′s exopthalmometer, conjunctival injection, and mild chemosis with mild exposure keratitis. Pupils were isocoric, round, reactive to light and there was no relative afferent papillary defect. Visual acuity was 6/60 in the right eye and finger counting (at 1 meter) in the left eye. Fundoscopy showed bilateral optic disc pallor. Intraocular pressures were 17.3 mm Hg. A provisional diagnosis of Grave′s opthalmopathy was made. Investigations revealed a normal complete blood count, kidney and liver function tests. Urine analysis was within normal limits. ECG, X-ray chest, abdominal ultrasound, and thyroid function tests were normal. Collagen profile was within normal limits; Venereal Disease Research Laboratory (VDRL) and Anti-neutrophil cytoplasmic autoantibody (ANCA) were negative.

**Figure 1:**
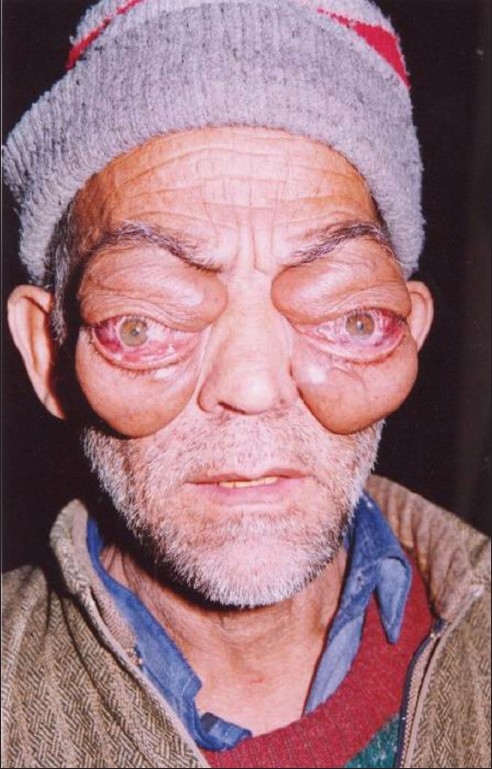
Proptosis of both eye balls

X-ray skull showed diffuse enlargement of the periorbital tissue on both sides. Computed tomography (CT) scan of the orbit showed bilateral proptosis (29 mm from orbital margin, Figures 2 and 3) with bilateral symmetric hypertrophy of all extraocular muscles and tendons. Both optic nerves were thickened. There were no skeletal changes or enhancing lesions on postcontrast study.

**Figure 2 and 3:**
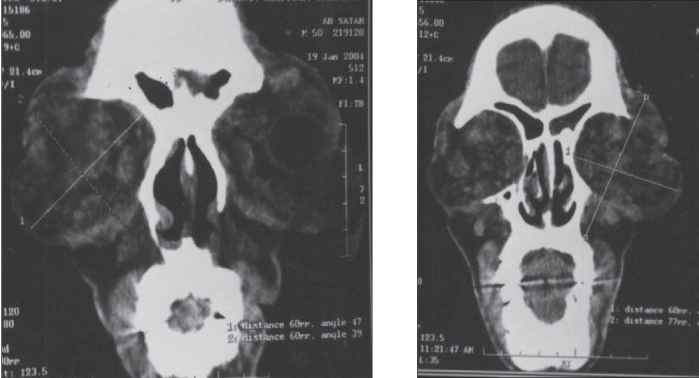
Computerized tomographic scan of the orbit with bilateral symmetrical proptosis (29 mm from orbital margin) and with bilateral symmetrical hypertrophy of the extra ocular muscles and tendons with thickening of optic nerves. There is neither bony erosion nor a signifi cant lesion on postcontrast study

Orbital magnetic resonance imaging (MRI) revealed bilateral diffuse infiltration of orbital fat, thickening of optic nerves, and enlarged lacrimal glands. The extraocular muscles were diffusely enlarged (tendons as well as muscle bellies in a tubular configuration) [Figure 4]. The perineural optic sheath complex was involved. Magnetic resonance angiography (MRA) revealed no abnormality related to orbit.

**Figure 4:**
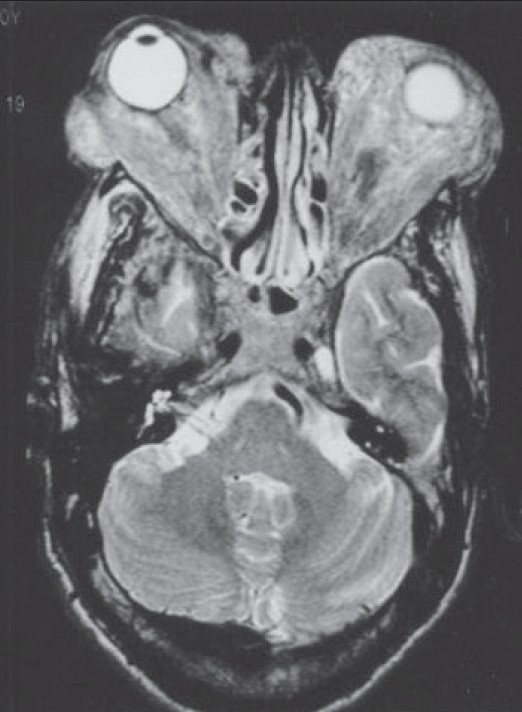
The orbital MRI reveals bilateral advanced proptosis with diffuse infiltration of orbital fat, obliteration of optic nerves, extraocular muscles with fi xation of intraorbital structures and enlarged lacrimal glands. Extraocular muscles show diffuse enlargement (both tendons as well muscle bundles enlarged in a tubular confi guration), with involvement of perineural optic sheath

## Tissue biopsy

**Figure 5:**
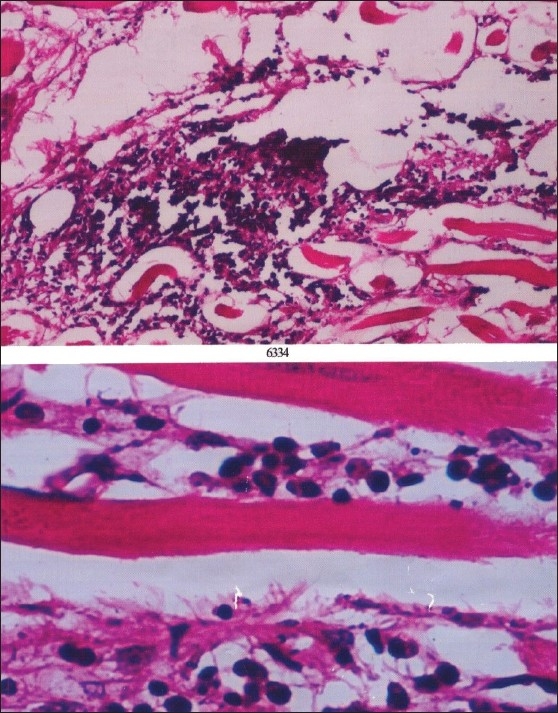
Microscopic examination of extra orbital tissue shows focal loose nodular collections of mononuclear inflammatory cells in the interstitium of skeletal muscle associated with fibrosis and fat infiltration with absence of Ganglion such as cells, atypical cells, specific granulomas or phagocytic cells

A tissue biopsy was taken and examined by four pathologists. The biopsy reports were consistent with the diagnosis of orbital pseudotumor [Figure 5].

## Hospital course

**Figure 6:**
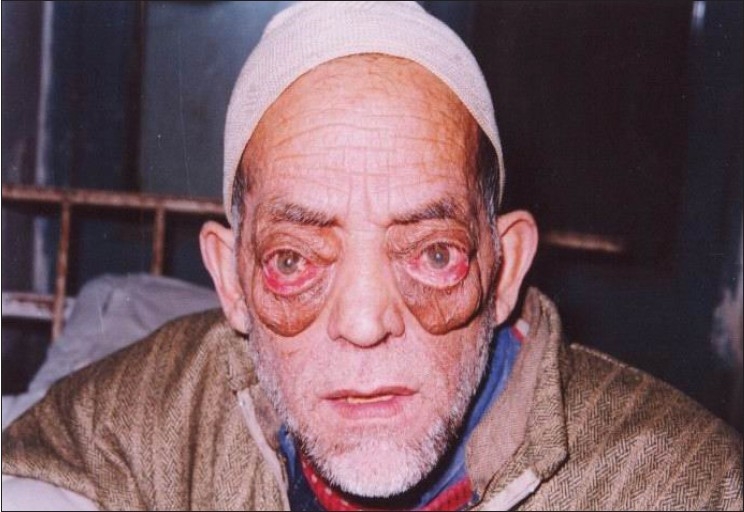
Response of the patient after corticosteriod treatment with decreased proptosis, decreased lid edema and conjunctival injection after eight weeks of treatment

The patient was put on oral predinisolone 100 mg daily. He responded promptly to the treatment with reduction of the proptosis, lid edema, and conjuctival injection and improvement of ocular motility and visual acuity [Figure 6]. Steroids were tapered over after a six-week period under close follow-up. Examination after eight weeks revealed that proptosis had reduced to 25 mm on right and 26 mm on left side. The patient is presently in remission.

Orbital pseudotumor also known as IOIS is a rare disease, [8,9] characterized by an inflammation of unknown cause occurring within the orbit simulating a neoplasm. Patients commonly present with an abrupt onset of diffuse or localized periocular pain associated with diplopia, restricted ocular motility and swelling and redness of the eyelids. [2,8,9] Unilateral presentation is typical but bilateral cases have been described earlier. [6] In a minority of patients, the presentation may progress over weeks (subacute) or insidiously over a period of months (chronic), as in our patient. General symptoms such as malaise, headache or nausea may accompany the ocular symptoms. Some unusual clinical presentations of orbital pseudotumor include temporal arteritis [10] and cluster headache. [11]

The pathogenesis of IOIS remains elusive, but several lines of evidence point to an immune mediated process as a likely underlying mechanism. [2,6,12]

The signs of IOIS are usually nondiagnostic being wide and diverse. Also, the histological picture is variable ranging from a diffuse polymorphous cellular infiltrate to the atypical granulomatous inflammation and infiltrative sclerosis. [2,6,13] In the present patient, an inflammatory infiltrate composed of mononuclear cells, lymphocytes and plasma cells interspersed with a variable amount of fibrovascular tissue, was seen on pathological examination. Several histopathological classification schemes have been proposed. To date, however, none of the numerous proposed classification schemes have been universally accepted as being definitive. [6] The radiological findings are characterized by inflammatory changes in various intraorbital structures; such as the globe, lacrimal gland, extraocular muscles, orbital fat, and optic nerve. [2,14]

MRI in our patient showed involvement of both lacrimal glands, which is the orbital structure involved in pseudotumor most frequently. [2,7] Enlargement of extraocular muscles and tendons also occurs frequently, [2] as seen in this patient on CT scans and MRI. The tendons enlarge with the muscle bundles and lead to a tubular configuration, which is in contrast to thyroid opthalmopathy, in which muscles reveal a spindle shaped configuration with normal tendons. [2,7] Pseudotumor may manifest as a diffuse infiltration in the orbital fat, enveloping the globe and surrounding the optic nerve sheath complex, which was also seen in this patient.

IOIS shows rapid response to systemic corticosteroid treatment. [2,6,7] Steroids are the established primary treatment for orbital pseudotumors. [15] They are administered for several months to ensure remission. Response to steroid therapy was demonstrated in this case also. Radiotherapy may be used in patients who fail to respond to steroids or who have a rapidly progressive course. [6,7,16] Many studies have shown a very high recurrence and low cure rate in patients treated with corticosteroids. [15,16] Alternative modalities, including radiotherapy and use of chemotherapeutic agents such as cyclophosphamide, methotrexate and cyclosporine, [7] have been tried with varying claims and deserve to be explored further.

To conclude, orbital pseudotumor is an uncommon, mostly unilateral disease with subacute or chronic onset. Inflammatory changes can be demonstrated on orbital imaging and good visual prognosis can be expected in most cases. Orbital MRI is the most important diagnostic test. Biopsy is usually not performed at presentation [7] because of the risk of damage to vital structures in the orbit. However, we feel that a biopsy should be taken to rule out any other underlying pathology.
